# Social acceptance of nursing during the coronavirus pandemic: COVID‐19 an opportunity to reform the public image of nursing

**DOI:** 10.1002/nop2.1267

**Published:** 2022-06-14

**Authors:** Vahid Zamanzadeh, Majid Purabdollah, Mostafa Ghasempour

**Affiliations:** ^1^ Department of Medical Surgical Nursing, Faculty of Nursing and Midwifery Tabriz University of Medical Sciences Tabriz Iran; ^2^ School of Nursing and Midwifery Tabriz University of Medical Sciences Tabriz Iran

**Keywords:** COVID‐19, nursing, public image, social media

Dear Editor

Nursing professionalism has been an important issue in the history of nursing and in recent decades significant progress has been made in this direction. One of the most important effective features in nursing professionalism is the public image and social acceptance towards nursing. Since Florence Nightingale, the public image of nursing has always been one of the major challenges of the nursing profession (Moore et al., 2019). The image of nursing is closely related to the identity and role of nurses and their cultural background and affects their clinical performance, job satisfaction, quality of care, social status, economic value and career development (Kaur Pushpinder & Rawat, 2017).

The image of nursing has changed over time; from angel and servant in 1919 to a weak and feminine image in the years 1920 to 1929 and the following decades to subordinate and receiver of doctors' orders, and it has finally, morphed into the image of a caregiver in recent decades (Girvin et al., 2016). Findings show that the public image of nursing is a multidimensional, all‐inclusive, paradoxical, dynamic and complex concept and it is influenced by nursing stereotypes. In previous studies, 34 different nursing stereotypes have been identified, some of which include angels of mercy, the doctor’s handmaiden, battle axe and sexy nurse. It should be noted that most of the identified stereotypes have negative connotations for the nursing profession and the media have played a key role in the lingering effects (Girvin et al., 2016). Furthermore, Rezaei‐Adaryani et al. (2012) summarized the undesirable social perception of nursing in terms such as gender stereotypes, being a physician subordinate, having lower academic standards, harsh working conditions, low income and limited job opportunities for career advancement. These negative stereotypes can lead to frustration, low self‐esteem and disruption of nurses’ professional and social identities, creating a repressive environment for enhancing the nursing profession (Fontanini et al., 2021).

There are many factors associated with the public image of nursing. Important factors include media, newspapers, magazines, poor communication, invisibility, nurses' self‐image, behaviours, clothing style, gender issues and professional organizations (Elmorshedy et al., 2020; Rezaei‐Adaryani et al., 2012). One of the factors that is less known but effective in creating and strengthening the public image of nursing is the occurrence of disasters such as earthquakes, tsunamis and emerging diseases such as severe acute respiratory syndrome (SARS), Middle East respiratory syndrome‐related coronavirus (MERS), Ebola, coronavirus and the role of nurses in managing these crises. It should be noted that there are no validated treatments for many new emerging infectious diseases such as SARS, MERS and COVID‐19 (Wibawa, 2021). The main strategies are symptomatic and supportive care, such as keeping vital signs stable, maintaining oxygen saturation and treating complications, such as secondary infections or organ failure (McGillis Hall & Kashin, 2016; Wibawa, 2021; Wu et al., 2020). Although we are 2 years into the Covid19 pandemic, due to the evolving nature of the virus and the emergence of new variants such as Delta and Omicron, additional studies on standard drug treatments are still underway (Cascella et al., 2022).

The many challenges and problems that COVID‐19 has created worldwide have received a great deal of media attention. The general public relies heavily on the media as well as government and global health organizations for accurate and comprehensive information in times of health crises. Consequently, the media have covered more news and images related to nurses in response to COVID‐19. The results show that despite a threefold increase in media coverage of nursing between 18 March and 18 April 2020 (coinciding with the outbreak of COVID‐ 19) compared to the same period last year, the stereotyped image of nursing, such as the Angels of Mercy with wounded faces, national heroes, self‐sacrifices shown to the public and even the use of military terms such as war, the battle on the front lines and death on duty, has become commonplace in both media and political discourses (Bennett et al., 2020). However, the use of these military concepts has recently been questioned. Comparing pandemics to war is both dangerous and wrong, since pandemics require collective, focused and coordinated responses, while wars divide the population (Fontanini et al., 2021; Varma, 2020).

Thus, the conflict between the nursing profession and stereotypical public images remains. In this regard, the results of the study of McGillis et al. showed that the media in the early stages of the Ebola outbreak compared nurses to Florence Nightingale and emphasized the traditional image of nurses as heroes caring for Ebola patients. Over time as Ebola spread outside the African continent, and medical and nursing practices were not successful in controlling Ebola, media stories began to cast a guilty image of nursing. In these reports, little attention has been paid to describing the role of nurses, their knowledge and expertise in resolving public health crises (McGillis Hall & Kashin, 2016).

For a long time, nurses have rarely been in the headlines. Even in crises like the COVID‐19 pandemic, based on an analysis of the front pages of Italian and Spanish newspapers to determine how the COVID‐19 crisis had been covered, healthcare providers are reported in only 6% of cases (Fontanini et al., 2021). A global health crisis such as COVID‐19 provides a unique opportunity for nurses to appear in the media, speak directly to the media, clarify information, and change the public perceptions of their professional roles in the healthcare system. The results of studies in China (Zhang et al., 2021) and Italy (Fontanini et al., 2021) showed that although part of the society accused nurses of negligence in the care and the cause of the spread of the pandemic, generally, the role and practice of nurses in the COVID‐19 pandemic has had a positive effect on elevating the public image of nursing. Presently, it seems that promoting public awareness about the roles of nurses in healthcare systems, especially in the face of crises such as COVID‐19, can be effective in removing the stereotypical image of nurses. It is also possible to create a new public image of the nursing profession in the community as a profession that requires higher education, specializes in its work and is independent of other medical professions.

For this purpose, nurses themselves should try to use the platform created in new social media such as YouTube and social networking platforms to demonstrate the unseen aspects of their profession; and combine their lesser known professional roles with their traditional roles to create a more professional, more valuable, nobler image to enhance the quality of care. Results of a study show that nurses who suffer from severe stress and traumatic experiences caused by COVID‐19 are more likely to use social media to report the problems, overworking and concerns over their own physical symptoms, rather than mentioning their professional issues and specialized roles in the COVID‐19 pandemic on social media (Fontanini et al., 2021).

In general, if we aim to turn the positive attention and discourse created about nursing into action, we should make the leadership and impact of nursing more visible in the public eyes. Governments and nursing leaders should provide the necessary infrastructure to recognize the professional and specialized roles of nurses in promoting health and strengthening healthcare systems by investing and developing training and counselling programmes with the active participation of nurses at various levels of society (Bennett et al., 2020).

## CONCLUSION

Although nurses have suffered and experienced hardships during the COVID‐19 pandemic, this crisis can be a suitable opportunity for their personal and professional development. This pandemic could provide a stronger voice for introducing the nursing profession, influencing healthcare policies and future nursing practices. It should be noted that the opportunity created, especially in the context of cyberspace and social media, should not be limited to narrating the problems, sufferings, shortages and workload of nurses, considering these issues will be forgotten after the pandemic subsides. Instead, this opportunity should be used by nursing managers and nurses themselves in order to reform misconceptions, provide a more professional voice for nurses in influencing future healthcare policies and practices and pave the way for reforming the public stereotypical image of nursing.

## CONFLICT OF INTEREST

The authors report no conflict of interest.

## Data Availability

The data that support the findings of this study are available from the corresponding author upon reasonable request.
